# The Effects of Supplemental Zinc and Honey on Wound Healing in Rats

**Published:** 2011

**Authors:** Ghasem Sazegar, Attarzadeh Hosseini Seyed Reza, Effat Behravan

**Affiliations:** 1 *Department of Anatomical Sciences and Cell Biology, Faculty of Medicine, Mashhad University of Medical Sciences, Mashhad, Iran *; 2 *Ferdowsi University of Mashhad, Iran*; 3 *Medical Toxicology Research Center, School of Pharmacy, Mashhad, University of Medical Sciences, Mashhad, Iran*

**Keywords:** Honey, Spectrophotometry, Tensile strength, Wound healing, Zinc Sulfate

## Abstract

**Objective(s):**

Clinicians have long been searching for ways to obtain "super normal" wound healing. Zinc supplementation improves the healing of open wounds. Honey can improve the wound healing with its antibacterial properties. Giving supplemental zinc to normal rats can increase the wound tensile strength. This work is to study the concurrent effects of zinc and honey in wound healing of normal rats.

**Materials and Methods:**

One hundred and seventy two young rats were randomly divided into four groups: control, zinc-supplement, applied honey, zinc-supplement and applied honey. Two areas of skin about 4 cm² were excised. The wound area was measured every 2 days. After 3 weeks, all animals were killed and tensile strength of wounds, zinc concentration of blood and histological improvement of wounds were evaluated. The results were analyzed using two-way ANOVA and the mean differences were tested.

**Results:**

It was found that honey could inhibit the bacterial growth in skin excisions. The tensile strength was increased significantly in the second to fourth groups at 21st day (*P< *0.001). Also there was a significant increase in tensile strength at the same time in the fourth group. The results of the histological study showed a considerable increase in the collagen fibers, re-epithelialization and re-vascularization in the second to fourth groups.

**Conclusion:**

The results of the present study indicate that zinc sulfate could retard re-epithelialization, but when used with natural honey (administered topically) it could have influent wound healing in non-zinc-deficient subjects as well.

## Introduction

Wounds are prevalent clinical problems and a burden to many patients, resulting in pain, discomfort, longer hospital stay, and considerable economic costs for the healthcare system (1). Wounds are either acute or chronic, and can result from venous or arterial insufficiency, diabetes, burns, trauma, chronic pressure or surgery (2-4).

Clinicians have long been searching for ways to obtain "super normal" healing of wounds using different models (5-7). These investigators found thiamine (vitamin B1) (8), pantothenic acid (vitamin B5) (3, 9,10) riboflavin (11), ascorbic acid (vitamin C) (12,13) vitamin A(14), cortisone (15,16), vitamin E (17,18), copper (19), manganese and silicone (19-22), and CO_2_ laser (23) effective on wound healing process. Among them, one managed to show that zinc supplementation would result in the improved healing of granulating open wounds (24-28). On the other hand, negative results have also been reported (29). 

Sanstead *et al* (24), Lansdown (26) and Agren (27) all have shown that zinc deficiency causes animals to heal poorly, while zinc have the potential to correct the abnormality. Other investigators attempted to show that giving supplemental zinc to an animal without zinc deficiency had been helpful to healing (30). 

Other studies showed that, with its antibacterial properties, honey can improve the wound healing (31,32).

Subrahmanyam reported that use of honey as a dressing in burned injuries rendered the wounds sterile and the healing process rich (33,34). Bergman *et al *(35), Yilmaz *et al *(36), Molan (32), Cooper *et* *al* (37), Tan *et al *(38), Obasieki-Ebor *et al *(39) all have shown that honey inhibited the growth of several organisms responsible for wound infections and accelerated the wound life span.

Ebrahimi *et al* showed that giving supplemental zinc to normal rats can increase the wound tensile strength, but unfortunately it can delay the wound span (28). It is the purpose of this work to study the concurrent effects of zinc and honey on wound healing of rats with no zinc deficiency.

Tensile strength, histological sections, photographic actual wounds measurements, determination of serum zinc level with atomic absorption, and bacterial growth, were used to measure healing. 

## Materials and Methods

One hundred seventy two (29) young Albino N-Mary rats with an average weight of 200 grams were studied. All animals were fed a standard pellet diet and were given water *ad libitum*, each housed in individual compartment of plastic cages. Cases were supplied to the containers at atmospheric pressure; the temperature was maintained at 25 to 27^ ◦^C with humidity between 70 and 90%.

The animals were randomly divided into four groups: first group: controlled with above regimen, second group: orally zinc-supplement with above regimen plus 36.3 mg zinc sulfate (8.25 mg zinc) a day, third group: applied honey with above regimen plus topically 10 ml natural honey twice a day and fourth group: orally zinc-supplement and applied honey with above regimen plus 36.3 mg zinc sulfate (8.25 mg zinc) a day and topically 10 ml natural honey twice a day.

**Figure 1:**
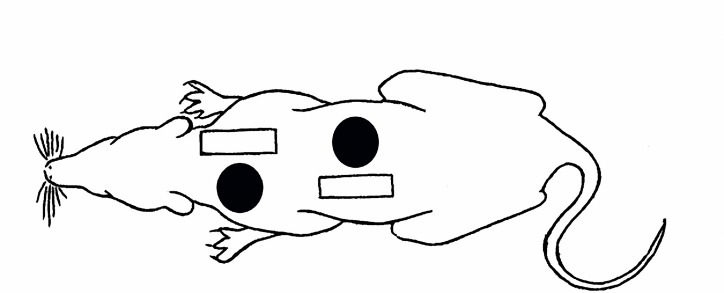
Diagram showing placements of excisions.

All animals were anesthetized with thiobarbital (Nesdonal g) 0.2 ml/100 g body weight administered intra peritoneally and hair was removed from the back with clippers. Operation was performed under clean but not strictly sterile conditions. Two areas of skin about 4 cm² were excised, one wound made cephalad, and the other caudad (Figure 1). Betadin (povidin iodine) was applied to each wound to prevent infection. All wounds were made by the same surgeon and in the same environment. After 3 weeks, all animals were anesthetized and killed with ether.

***Measurement of wound area***

**Figure 2:**
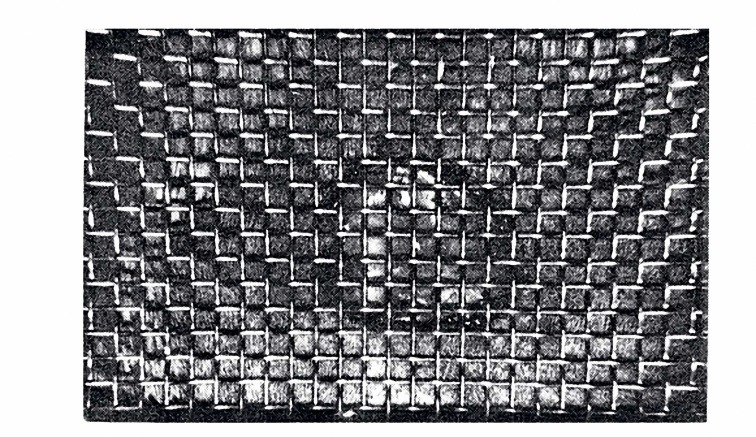
Measurement of surface area of dorsal open wounds in rats by the photographic grid method.

The wound area was measured every two day until healing had taken place. The photographic grid method of measuring wound area was used. In this method every two day a wire grid was placed over the wound. It was then photographed, and the number of small squares over the non epithelialized area were counted in a high resolution (Figure 2).

***Tensiometeric method***

The skin of the back, including the wounds, was shaved, excised (5 cm long and 1 cm in diameter) and immediately transferred to a Petri dish of normal saline and stretched to its normal size. The maximal strength of this wound strip cut perpendicular to the wound alignment was measured using a materials testing machine (Zwic ®).

***Chemical methods***

Hemoglobin concentration was measured by the cyanmethemoglobin method. 

***Zinc determination***

A blood sample was obtained from each rat by intra cardiac puncture prior to sacrifice to determine the plasma zinc level using an atomic absorption spectrophotometer (,England)

***Histological examination***

A biopsy was taken for histological examination after fixation in 4% formaldehyde and staining with Van Gieson-Elastin or Hematoxilin-Eosin. All assessments were made with the histological slides coded to reduce subjective bias, and for quantitative assessment each slide was evaluated by a pathologist and received a score between 1 and 4. Score 1 means that the amounts of collagen fibers, re-epithelialization and re-vascularization in skin are a little more than control skin. Score 4 means considerable increase in the collagen fibers, re-epithelialization and re-vascularization, and score 2 and 3 were in the middle of the spectrum. 

***Statistical analysis***

Data are expressed as mean±SEM. Statistical analysis was performed using two-way ANOVA followed by Tukey–Kramer post-hoc test for multiple comparisons. The *P*-values less than 0.05 were considered to be statistically significant.

## Results

All wounds healed without infection in four groups. In this study, infection means the concentration of bacteria signs including red skin around the wound, discharge containing pus, swelling, warmth, foul odor, and fever.

 Bacterial growth was studied in early granulation tissue of all groups. The findings show that bacterial growth in full-thickness skin excisions was inhibited with honey in third and fourth groups. 

 The average weight gain in the two non-zinc supplemented groups showed no significant difference compared to the other groups. In the first and third groups, the weight increased from 202.2±12.3 g (mean±SEM) at the beginning of the experiment to 219.8±8.8 g at the end. In the second and fourth groups, which received zinc supplements, the body weight increased from 198.7±10.5 g to 221.5±11.8 g during the experiment. 

**Figure 3:**
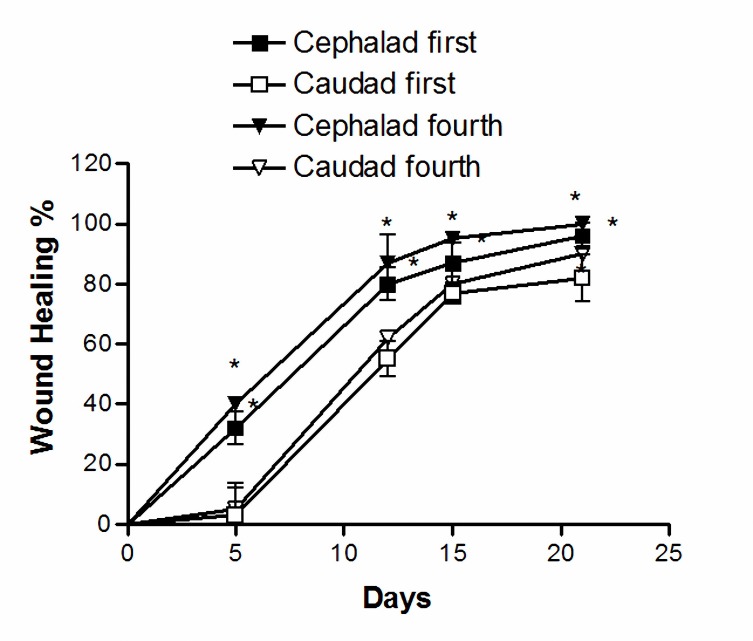
Progression of healing of first and fourth groups cephalad and caudad open wounds. The first group received standard diet, and the fourth group received oral zinc sulfate (36.3 mg) and applied topically 10 ml honey. The wound healing was measured with a wire grid over the wound and the numbers of small squares were counted. In all cases of the first and fourth groups, the cephalad wound healed at a significantly faster rate compared to the caudad wound upto day 21 postoperative. Values are mean±SEM (n= 3). *P< 0.05, two-way ANOVA followed by Tukey–Kramer test).

**Figure 4.**
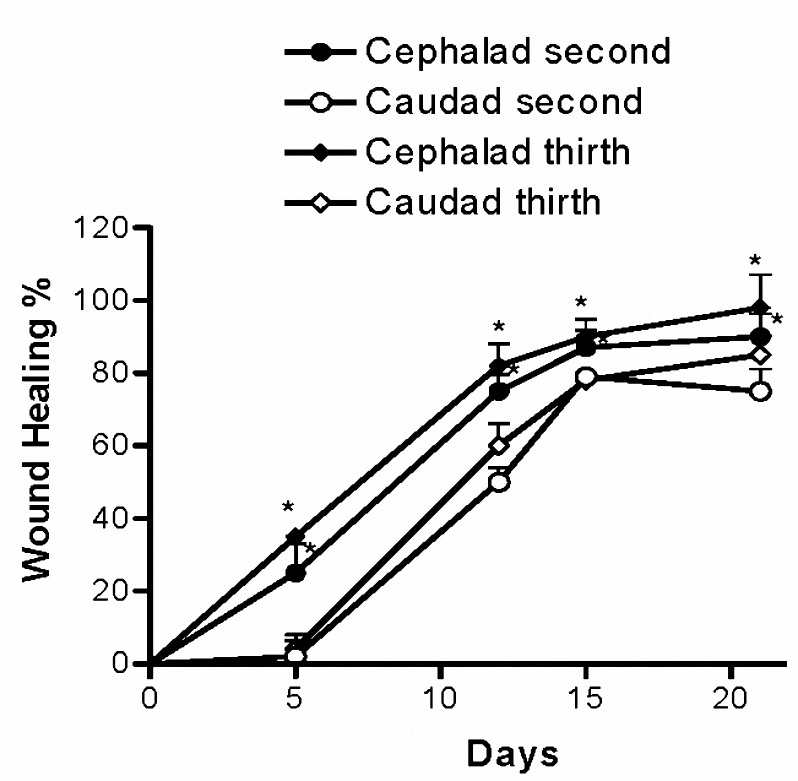
Progression of healing of second and third group´s cephalad and caudad opens wounds. The second group received oral daily 36.3 mg zinc sulfate and the third group received twice daily topical 10 ml honey. The wound healing was measured with a wire grid over the wound and the numbers of small squares were counted. In all cases of the second and the third groups, the cephalad wound healed at a significantly faster rate compared to the caudad wound upto day 21 postoperative. Values are mean±SEM (n= 43). *P< 0.05, two-way ANOVA followed by Tukey–Kramer test).

 Figures 3 and 4 shows the progression of wound healing in the four studied groups. In all cases of the four groups, the cephalad wound healed at a significantly faster rate compared to the caudad wound up to day 21st postoperative day (*P< *0.05). Although the second group showed a considerably slower rate of closure, no significant difference in the healing rate was observed at any point in time between the first, third and fourth groups cephalad or caudad wounds (Figure 3, 4). 

**Figure 5:**
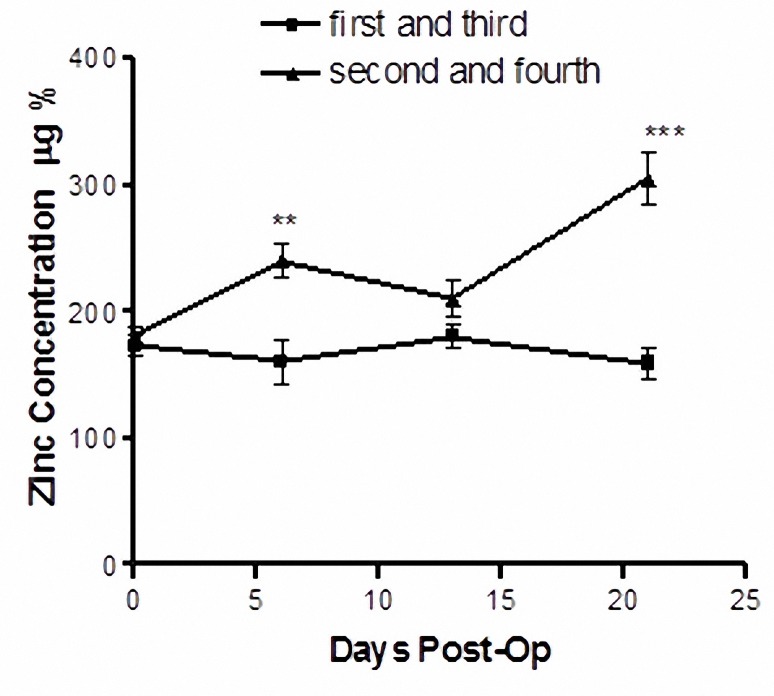
Zinc level in the rats blood serum. The blood zinc concentration in non zinc supplemented animals (first and third groups) at 21st day post operative was 172.2±8.4 µg/100 ml (mean±SEM) and in zinc supplemented animals (second and fourth groups) at the same time was 302.8±12.8 µg/100 ml. Values are mean±SEM (n= 43). **P< 0.01, ***P< 0.001, one-way ANOVA followed by Tukey–Kramer test).

 In the Figure 5 the plasma zinc levels are shown for the rats in the non-diet zinc supplemented groups and diet zinc supplemented groups. There was a significant increase of 85% after 3 weeks of zinc administration (*P*< 0.05). 

**Figure 6.**
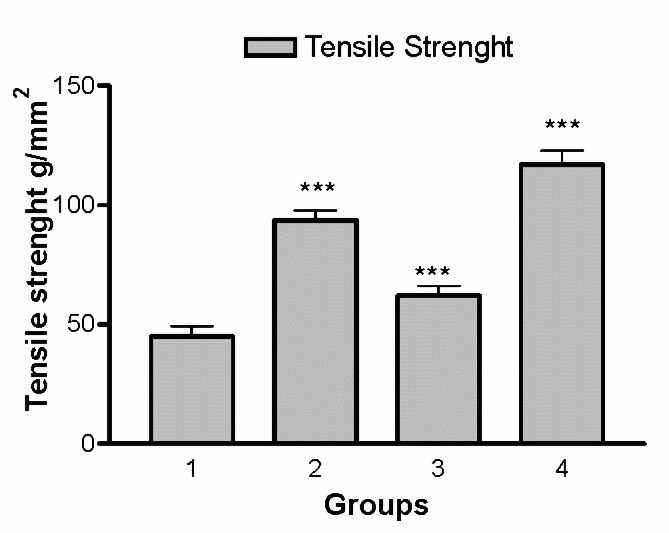
Tensile strength of excised wounds in the four studied groups. There is a significant increase in tensile strength at the 21st day in the second, third and fourth groups compared to the first group ***P< 0.001, one-way ANOVA followed by Tukey–Kramer test).

 In the fourth group, the tensile strength was measured in 21 days after wounding (Figure 6). The test groups (second, third and fourth groups) showed a significant increase in tensile strength in the 21st day when compared with control (first) group (F= 2107, *P< *0.001), (Figure 6). Also there was a significant increase in tensile strength at the same time in the fourth group when compared with the second group, and in the second group when compared with the third group (*P< *0.01). 

**Figure 7.**
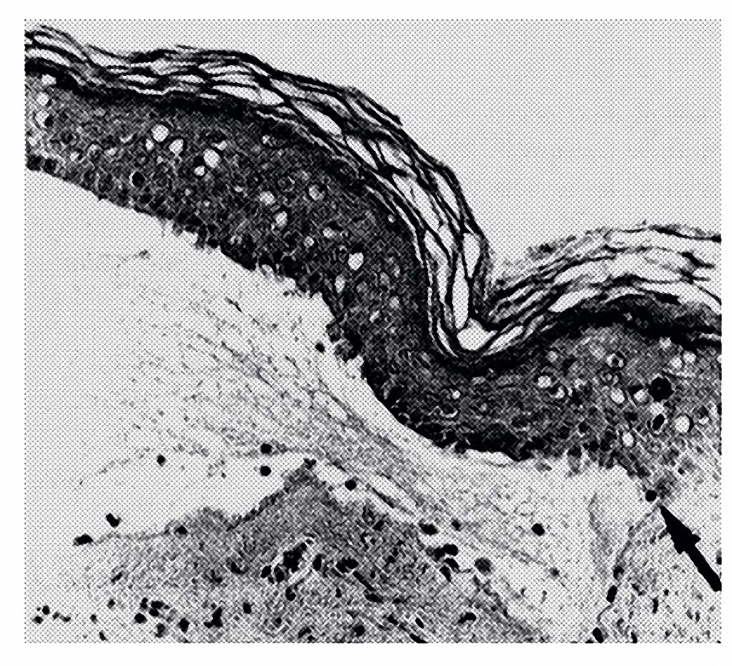
The edge of a blister formed on skin in fourth group. The site of separation between dermis and epidermis is marked with an arrow. The blister fluid is rich of fibrin but essentially free of inflammatory cells. Restoration of surface epithelium over wound which extends down from base of depressed area or pocket is seen. Hematoxylin-Eosin.x400.

**Figure 8.**
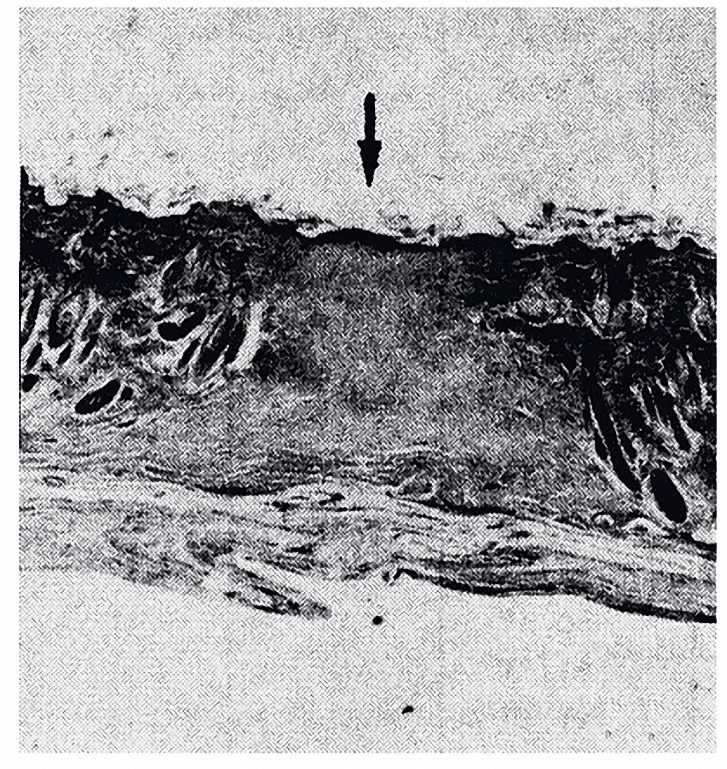
Cross section of skin in the first group. The dark band at the top of the photo is the epidermis. The dense material above the epidermis is a scab. The wound is closed at the level of full thickness skin. Notice that the epidermis above and adjacent to the wound site is thicker than the epidermis further away. The site of wounding is marked with an arrow. Hematoxylin-eosin.x400.

 In the present study, all the cephalad wounds were studied by light microscopy () to investigate the collagen fibers, re-epithelialization and revascularization. The results of the histological study showed a considerable increase in the collagen fibers, re-epithelialization and re-vascularization between second, third and fourth groups when compared with the first group (f= 20.33 and *P< *0.001), (Figures 7, 8).

## Discussion

This study showed that supplements of zinc sulfate administered orally when used in combination with honey, may have the effect of enhancing the growth of granulation tissue, promoting the vascularization of the wound site, and/or stimulating the process of epidermal migration and increase of wound tensile strength.

In the rat, zinc toxicity is characterized by depressed growth and anemia (40). When zinc sulfate was administered orally at the rate of 36.3 mg/day in the present study, there was no evidence of such toxic effects as judged by weight and hemoglobin measurements at the beginning and end of the experiments.

 The rate of new collagen deposition as determined by Muscará *et al* measurements (41) reaches a maximum between the fourteenth and twenty first days and remains elevated through the tenth week, and in the present study all animals were killed after 3 weeks of surgery. 

 Zinc is a trace mineral that is a component of many enzymes, including DNA and RNA polymerases, and is required for protein synthesis, DNA synthesis, mitosis, and cell proliferation. Approximately 300 enzymes need zinc for proper functioning; many of these zinc-dependent processes, such as collagen synthesis and cell division, are required for wound healing (42). 

 Zinc acts as a co-factor for enzymes involved in wound healing, the most notable lysyl oxidase, which catalyzes the cross- linkage of collagen (43). The importance of cross- linking for the mechanical strength of wounds was demonstrated by topical administration of ß-aminopropionitril, which irreversibly inhibits lysyl oxidase (44).

 Lysyl oxidase catalyses the formation of covalent cross-links by formation of reactive aldehyde groups on the collagen molecules. Although lysyl oxidase is a copper- dependent enzyme, its activity is to decrease granulation tissue of zinc- deficient rats (45). Thus a possible explanation for the decreased breaking strength is diminished cross- linking in zinc deficiency. But other factors, such as the interaction with proteoglycans and the structure and orientation of collagen fibers, probably influence the wound strength as well (30,46). Ultra structural studies of 2-week skin incisions in rats (27,44) revealed no morphologic difference between zinc- deficient and control animals in the orientation or thickness of the collagen fibers.

Previous experiments proved increase accumulation of wound collagen in animals treated with topically applied natural honey (31, 32,46).

 Honey provides antibacterial properties (31,32) and contains enzymes such as catalase which aid in the healing properties (46). Honey also rapidly debrides wounds and removes malodor, and its anti-inflammatory activity reduces edema and exudates, and prevents or minimizes hypertrophic scarring (32). 

 Honey has a remarkable effect on the process of re-vascularization of the wound, within 15 min-6 hr after application, the treatment wound takes an intensely bright red color, visual evidence of the fact that new blood vessels are being formed in the region, and that normal circulation is returning to the site. 

Although it is part of the normal healing process that re-vascularization will eventually occur, the speed with which new blood vessels return to the damaged tissue when treated with zinc and honey is unexpectedly faster than that observed with honey alone. Similar surprising effects are seen in the development of granulation tissue.

 This study showed that, although zinc sulfate can increase tensile strength in wounding skin, it causes delay in the rate of closure of skin defects. This is in accordance with the findings of Ågren in1990 (29) and Ebrahimi *et al* in 1996 (28). Honey, when used alone, shows a remarkable and unpredicted effect on control of bacterial infection on damaged skin, and when used with zinc sulfate, can correct the effects of zinc sulfate as shown in the fourth group.

## Conclusion

The results of the present study indicate that when zinc is administered orally as zinc sulfate it can retard re-epithelialization, but, when used with natural honey (administered topically), can favorably influence wound healing in non-zinc-deficient subjects as well. Since re-epithelialization is an important mechanism in the closure of ulcers these results taken together imply that topically applied honey and orally applied zinc might increase the healing rate not only in animals with low serum zinc level but in animals with normal zinc status as well.

Although topical honey and oral zinc sulfate can promote re-epithelialization, diminish inflammation and reduce bacterial growth in wounds, the exact mechanisms by which honey and zinc exerts this effects are still not clear. The mechanisms are probably complex due to the interaction of zinc and honey with many enzyme systems and with biomembranes. 
